# Spider venom components decrease glioblastoma cell migration and invasion through RhoA-ROCK and Na^+^/K^+^-ATPase β2: potential molecular entities to treat invasive brain cancer

**DOI:** 10.1186/s12935-020-01643-8

**Published:** 2020-12-17

**Authors:** Natália Barreto, Marcus Caballero, Amanda Pires Bonfanti, Felipe Cezar Pinheiro de Mato, Jaqueline Munhoz, Thomaz A. A. da Rocha‐e‐Silva, Rafael Sutti, João Luiz Vitorino-Araujo, Liana Verinaud, Catarina Rapôso

**Affiliations:** 1 Faculdade de Ciências Farmacêuticas, Universidade Estadual de Campinas (UNICAMP), Campinas, São Paulo, 13083-865 Brazil; 2 Departamento de Biologia Estrutural e Funcional, Instituto de Biologia, UNICAMP, São Paulo, Brazil; 3 Faculdade Israelita de Ciências da Saúde Albert Einstein, São Paulo, SP Brazil; 4 Faculdade de Ciências Médicas, Santa Casa de São Paulo, São Paulo, SP Brazil; 5 Disciplina de Neurocirurgia, Faculdade de Ciências Médicas da Santa Casa de São Paulo, São Paulo, SP Brazil

**Keywords:** *Phoneutria nigriventer*, Cytoskeleton, Cell adhesion, Cell morphology, Metastasis, Cancer therapy

## Abstract

**Background:**

Glioblastoma (GB) cells have the ability to migrate and infiltrate the normal parenchyma, leading to the formation of recurrent tumors often adjacent to the surgical extraction site. We recently showed that *Phoneutria nigriventer* spider venom (PnV) has anticancer effects mainly on the migration of human GB cell lines (NG97 and U-251). The present work aimed to investigate the effects of isolated components from the venom on migration, invasiveness, morphology and adhesion of GB cells, also evaluating RhoA-ROCK signaling and Na^+^/K^+^-ATPase β2 (AMOG) involvement.

**Methods:**

Human (NG97) GB cells were treated with twelve subfractions (SFs—obtained by HPLC from PnV). Migration and invasion were evaluated by scratch wound healing and transwell assays, respectively. Cell morphology and actin cytoskeleton were shown by GFAP and phalloidin labeling. The assay with fibronectin coated well plate was made to evaluate cell adhesion. Western blotting demonstrated ROCK and AMOG levels and a ROCK inhibitor was used to verify the involvement of this pathway. Values were analyzed by the GraphPad Prism software package and the level of significance was determinate using one-way analysis of variance (ANOVA) followed by Dunnett’s multiple comparisons test.

**Results:**

Two (SF1 and SF11) of twelve SFs, decreased migration and invasion compared to untreated control cells. Both SFs also altered actin cytoskeleton, changed cell morphology and reduced adhesion. SF1 and SF11 increased ROCK expression and the inhibition of this protein abolished the effects of both subfractions on migration, morphology and adhesion (but not on invasion). SF11 also increased Na^+^/K^+^-ATPase β2.

**Conclusion:**

All components of the venom were evaluated and two SFs were able to impair human glioblastoma cells. The RhoA effector, ROCK, was shown to be involved in the mechanisms of both PnV components. It is possible that AMOG mediates the effect of SF11 on the invasion. Further investigations to isolate and biochemically characterize the molecules are underway.

## Background

Gliomas are the most common primary intracranial tumors and are characterized by high morbidity and mortality [1]. Based on the degree of anaplasia, these tumors are divided into four grades, where grade I is considered benign, with favorable prognosis, and grade IV is associated with highly malignant tumors and includes glioblastoma (GB), the most devastating type of glioma [2, 3]. GB accounts for 80% of primary neoplasms of the brain (i.e. that originated from the tissues of the brain or immediate surroundings: glia, neurons, blood vessels, glands) and is one of the deadliest solid tumors of the central nervous system (CNS), leading to 225,000 deaths worldwide each year [4, 5].

Standard treatment usually includes surgery and chemotherapy with temozolomide [6]; However, studies have shown that at least 50% of patients do not respond to this chemotherapy, and even those who respond have low survival [7]. In fact, in the 1970s, the standard treatment protocol involved surgical removal of the tumor and radiotherapy, responsible for an average survival of 9 months. In the 2000s, the implementation of the use of temozolomide (TMZ) increased survival to 15 months [8]. Many potential drugs for the treatment of GB have been suggested, but the causes of failure are unknown [4]. One possible cause involves the blood–brain barrier (BBB), which may limit therapeutic efficacy by preventing most anticancer agents from being released from the blood vessels to neoplastic cells [9]. The fact that GBs are multi-driver tumors is another challenge. Asif et al. [10] demonstrated that genes with significant mutation detected in 17 GB patients included TP53, EGFR, PIK3R1, PTEN, NF1, RET, STAG2, IDH1, ATRX, MGMT methylation and others. Therefore, a multi-target therapy is necessary, making it more difficult to have a responsive medication. Another potential cause of treatment failure is that GB consists of cells with a high capacity to infiltrate healthy tissue, making complete removal difficult by surgery [5]. Therefore, it is increasingly necessary to focus on the development of new therapies against gliomas. Finding target-oriented molecular entities to regulate migration and invasion, preventing the infiltration and metastasis, has been a challenge in the antineoplastic pharmacology.

Natural products have greatly contributed to the history and panorama of new molecular entities. Patridge et al. [11], evaluated all new FDA (Food and Drug Administration) approved molecules, revealing that natural products and their derivatives represent more than a third of the new entities. Almost half of these are obtained/derived from mammals, a quarter from microbes and a quarter from plants. Arthropod venoms (mainly spiders and scorpions), on the other hand, are an underexploited source of new molecular entities. These venoms are an extremely complex and rich mixture of bioactive components. Its components have high affinity for multiple targets in the body and are currently being studied as potential prototypes of antineoplastic drugs based on optimized molecules [12, 13]. The venom of the South American spider *Phoneutria nigriventer* (PnV) (Ctenidae, Araneomorphae) has been shown to permeate the BBB and also has a particular response in astrocytes [14–17]. These studies lead to the hypothesis that investigation of the action of PnV and its purified toxins on glioma cells could be promising.

Based on these initial studies, our research group investigated the role of the crude PnV on viability, cell cycle, and migration of tumor cells [18]. This screening showed that the venom has anticancer effects mainly on human GB cell lines (NG97 and U-251). Therefore, considering that spider venoms are a complex mixture of molecules, it has become necessary to find the component(s) present in the venom responsible for these antineoplastic effects. In addition, the mechanisms behind these effects must be known. The present work aimed to investigate the effects of isolated PnV-fractions (F) and subfractions (SFs) on human GB cells, specifically on migration, invasiveness, morphology and adhesion. The most active SFs were selected and the involvement of RhoA-ROCK (Rho-associated protein kinase) and Na^+^/K^+^-ATPase β2 (glial adhesion molecule—AMOG) was evaluated. With these findings, the study aims to contribute to the development of new anticancer therapies, meeting the social demand for cancer treatment. The active peptides are being identified for further synthesis in order to contribute to the treatment of glioma and its subtypes, mainly the most aggressive ones.

## Methods

### Reagents and *Phoneutria nigriventer* venom (PnV)

All chemicals were obtained from Sigma Aldrich (St. Louis, MO), unless otherwise indicated. Two samples of lyophilized PnV were obtained by electrical stimulation of numerous adult spiders (males and females) (Sisgen #A551346). The quality and reproducibility of the venom were evaluated by high‐pressure liquid chromatography (HPLC). Lyophilized venom, fractions (F) and subfractions (SF) were stored at − 80 °C and dissolved immediately prior to use.

### Venom purification

The initial fractionation of the crude venom was performed by the Amicon Ultra Centrifugal Filter (#UFC801008; Thermo Fisher Scientific, Suwannee, GA). This procedure consisted of separating crude venom by molecular mass using molecular filters, generating three main fractions named: F1 (low weight, less than 3 kDa), F2 (intermediate weight, between 3 and 10 kDa) and F3 (high weight, more than 10 kDa). From these, experiments were conducted to select the most significant fraction, considering the antineoplastic effects; Then, F1 and F2 were chosen and F3 was eliminated. Further purification of F1 and F2 was carried out: reversed phase HPLC was performed using a Shimadzu VP-ODS column, 0.1% trifluoroacetic acid (TFA) as mobile phase and 90% acetonitrile 0.1% TFA as eluent. More purified components were obtained, named subfractions 1—12 (SF1—SF12).

### Cell culture

Human GB (NG97) cells were donated by a patient from the Hospital das Clínicas/Universidade Estadual de Campinas (HC/UNICAMP) and the cell line was established and characterized in a sequence of published studies [19–23]. After 4 passages from the defrosting, cells were seeded at a density of 1 × 10^4^ per cm^2^ in a 25 cm^2^ culture bottle and grown in Iscove’s modified Dulbecco´s medium (IMDM) containing 10% fetal bovine serum (FBS) and 100 UI/ml penicillin and streptomycin (pH 7.4) (Gibco). Cell culture was maintained in a humidified atmosphere at 37 °C and 5% CO_2_ until semi-confluence (about 90% of total surface area). For assays, cells were transferred after careful scraping to 24, 48 or 96-well plates (Corning Inc., New York, NY). All assays were performed in at least three independent experiments.

### ROCK inhibitor

In order to analyze the mechanism of PnV and its SFs on cell migration, morphology, and adhesion, cells were preincubated with 5 mM Y-27632 (Cayman Chemical Company—Michigan, USA; #10005583), a ROCK inhibitor. For these assays, the inhibitor was first reconstituted in dimethyl sulfoxide (DMSO) to a concentration of 10 mM and then diluted with IMDM to final concentration. GB (NG97) cells were incubated with the inhibitor for one hour prior the treatments. Subsequently, Y-27632 was re-placed and maintained during the treatment periods (0, 12, 24, 48 and/or 72 h) together with PnV and SFs. A control with Y-27632 only was performed.

### Scratch-wound healing migration assay

Cell motility was investigated by scratch-wound healing assay. After 90% confluence in a 48 well plate (2 × 10^5^ cells seeded per well), a scratch was made in each well with a 200 µl tip, followed by washing with serum-free medium to remove cells debris. The medium from each well was subsequently replaced and the cells were treated with PnV (14 µg/ml), fractions (F1, F2 and F3; 0.1, 1.0 or 10 µg/ml) and subfractions (SF1-SF12) (0.1 or 1.0 µg/ml); Controls remained in IMDM. The closure of the scratch area by cell migration was evaluated and photographed at various time points (0, 24, 48 and 72 h) by an inverted microscope (Nikon Eclipse TS100; Nikon, Tokyo, Japan), equipped with a camera and using the Nikon ACT-1 software package; or by the Cytation 5 Microplates Reader (BioTeK). Migration films (over 72 h period) were also recorded on Cytation 5.

### Cell proliferation assay—carboxyfluorescein succinimidyl ester (CFSE)

NG97 cells (1.0 × 10^6^) were labeled with CFSE probe (#C34554; Life Technologies Corporation, Eugene, OR) according to the supplier’s instructions and seeded in 96‐well plates (Corning, Inc., New York, NY). After 24 h of exposure to F1, F2 or F3 (1 μg/ml) (the controls were maintained in the medium) the cells were washed and the decay of the probe was measured. Data were described as proliferation index (PI), analyzed by FlowJo software (v7.6.5; Tree Star. Inc., Ashland, OR).

### Apoptosis–necrosis assay

NG97 cells were seeded in 24‐well plates (Corning, NY) at an initial density of 1.0 × 10^5^ cells per well and incubated at 37 °C for 72 h. The cells were then treated with F1, F2 or F3 (1 µg/mL), while the control cells were maintained in the medium. To determine the extent of apoptosis and necrosis, 1.0 × 10^5^ cells were stained with fluorescein isothiocyanate (FITC)–conjugated annexin V and propidium iodide (Pi), using the annexin V‐FITC apoptosis detection kit (#640,914; Biolegend, San Diego, CA) following the manufacturer’s instructions. A total of 10,000 cells were analyzed and cell apoptosis–necrosis was determined using the FACSVerse Cytometer and FACSuite system (BD Biosciences). The data were analyzed with FlowJo software (v7.6.5; Tree Star. Inc., Ashland, OR).

### MTT cell viability test

Thiazolyl Blue Tetrazolium Bromide (MTT), whose reduction by the dehydrogenase enzymes to insoluble formazan is associated with cellular activity, was used to determine if SFs would alter cell viability. NG97 cells were seeded in 96-well plates at an initial density of 5 × 10^5^ cells per well and incubated for 72 h at 37 °C for confluence. Then, cells were treated with PnV (14 µg/ml) and all twelve subfractions (SF1-SF12; 1.0 µg/ml) for 24 h, while control cells were maintained in medium. After removal of the treatments, MTT was added to each well and incubated at 37 °C for 4 h according to the manufacturer's protocol. Thereafter, acidified isopropanol was added to each well to solubilize the blue formazan crystals. Absorbance at 540 nm was determined on a Multiskan GO microplate spectrophotometer (Thermo Fisher Scientific, Inc., Waltham, MA, USA).

### Transwell invasion test

Tumor cell invasion was investigated using transwell inserts with 5 μm pore polycarbonate membranes (Corning Inc., Kennebunk, ME; #24417017). About 2 × 10^5^ cells in serum-free culture medium (IMDM) were seeded on top of each insert. Medium supplemented with 20% FBS was added to the bottom chamber of each well. Control cells were maintained only in serum-free culture medium; PnV (14 µg/ml) or SFs selected from scratch-wound healing results (SF1 and SF11—SFs with the best migration-impairing effect; 1.0 µg/ml) were added to the upper chamber and incubated for 12 and 48 h in a humidified atmosphere at 37ºC and 5% CO_2_. Some wells received the ROCK inhibitor prior and during the treatments, as described above. Unmigrated cells were removed from the upper surface using cotton swabs and migratory cells could be visualized at the underside of the membrane. For this, the cells were fixed with 4% paraformaldehyde (2 min), permeabilized with methanol (20 min), and stained with 1% Giemsa solution (15 min). Migrating cells were observed using an inverted microscope (Nikon Eclipse TS100; Nikon Tokyo, Japan) equipped with a camera and using the Nikon ACT-1 package software. Three inserts were evaluated by treatment in at least five fields randomly selected per membrane.

### Immunofluorescence and stress fiber visualization

Cell morphology and cytoskeleton were evaluated using phalloidin to selectively label F-actin and GFAP-immunolabeling (glial fibrillary acidic protein, an astrocyte marker). NG97 cells were seeded in 48-well plates (1 × 10^5^ cells per well) with IMDM. After 90% confluence, the medium was removed from the wells, which received a new medium added with PnV (14 µg/ml), SF1 or SF11 (1.0 µg/ml). Controls remained in IMDM. Some wells received Y-27632 (ROCK inhibitor) before and during treatments as described above. After 1, 12 and 48 h of treatments, cells were fixed with 4% paraformaldehyde (15 min), washed 3 times with phosphate buffered saline (PBS) and incubated with a permeabilization solution (0.1% Triton X100 in PBS) for 10 min at room temperature. The wells were washed with PBS and a blocking solution (1% bovine serum albumin—BSA plus 0.2% Tween 20 in PBS) was added for 1 h. The cells then received the phalloidin probe 1:200 (Sigma-Aldrich; #P5282) in dilution solution (0.3% BSA plus 0.1% Tween 20 in PBS) for 2 h at room temperature. The wells were washed with PBS and incubated overnight with anti-GFAP (1:500; Proteintech #16,825-I-AP) in dilution solution. The following day, cells were washed again with PBS and incubated with CY2-conjugated secondary antibody 1:1000 (Jackson Research; #111225144) for 1 h, followed by incubation with DAPI 1:1000 (Sigma-Aldrich; #D9542) (5 min) and then mounted with Glycerol: PBS (1:2). Cells were visualized by Cytation 5 (BioTeK) and analyzed using Gen 5 software, v. 3.04.

### Adhesion assay

Cell adhesion was verified using the CytoSelect™ Cell Adhesion Assay kit (Cell Biolabs, Inc.; #7251317), following the manufacturer's instructions. NG97 cells were seeded in 24-fibronectin coated well plate (2 × 10^5^ cells per well) and immediately exposed to PnV (14 µg/ml), SF1 or SF11 (1.0 µg/ml). BSA-coated wells were provided as a negative control. After 90 min (time was determinate by the manufacturer) of incubation with the treatments, each well was washed with PBS added with 2 mM CaCl_2_ and 2 mM MgCl_2_ (pH 7.4) and received the Cell Stain and Extraction Solution. Cells were then removed from each well and seeded into a 96-well microplate to measure absorbance (560 nm) on a Multiskan GO microplate spectrophotometer (Thermo Fisher Scientific, Inc., Waltham, MA, USA).

### Sodium dodecyl sulfate-polyacrylamide gel electrophoresis (SDS-PAGE) and western blot

After PnV (14 µg/ml), SF1 or SF11 (1.0 µg/ml) treatments for 5 h, cells were rinsed twice in PBS and immediately lysed for 20 min on ice in cold radioimmunoprecipitation assay (RIPA) (Cayman Chemical Company—Michigan, USA; #10,010,263) lysis buffer containing 10 ml 250 mM Tris–HCL, pH 7.6, 750 mM sodium chloride, 5% Tergitol (NP-40), 2.5% sodium deoxycholate and 0.5% SDS, supplemented with a protease inhibitor cocktail containing 10 mM sodium orthovanadate minimum 90% titration and 0,1 mg/mL aprotinin—bovine lung solution (Calbiochem, USA and Canada; #616399). The protein content was determined using Bradford protein assay (Bio-Rad, Hercules, CA, USA). Standards (0–1 mg/ml BSA) and samples were mixed with the reagent, incubated for 15 min at room temperature, measured at 595 nm using a Multiskan GO microplate spectrophotometer (Thermo Fisher Scientific, Inc., Waltham, MA, USA). All samples were analyzed for total protein content and 40 ug of total protein from each sample were loaded on each gel strip. β-actin was used as a loading control. SDS-PAGE was conducted using 8% (ROCK) or 12% (AMOG) Bis–Tris gels at 200 V for 50 min and the separated proteins were transferred at 30 V for 60 min to a nitrocellulose membrane (BioRad). The membranes were rinsed twice and the proteins were blocked with nonfat skim milk in TBS-T (0.1% Tris-buffered saline with 0.05% Tween 20, pH 7.4) for 60 min at room temperature. The membranes were probed using rabbit polyclonal anti-ROCK 1:500 (Proteintech Group Inc; #21,850–1-AP) or anti-ATP1B2, β2 subunit of Na^+^/K^+^-ATPase 1:500 (Proteintech Group Inc; #22,338–1-AP) and mouse monoclonal anti-β-actin 1:5000 (Sigma-Aldrich; #A5441). The membranes were then washed 4 × 8 min with TBS-T and incubated in a horseradish peroxidase (HRP)-conjugated secondary antibody, either goat anti-rabbit or donkey anti-mouse diluted 1:2000 (Proteintech Group Inc; #SA00001-1; #SA00001-2), followed by several washes with TBS-T. All primary and secondary antibodies were diluted in nonfat skim milk in TBS-T (3 and 1%, respectively). The antibody-bound protein was detected using an enhanced chemiluminescence kit (Super Signal, Pierce, Rockford, IL, USA) and visualized using GeneGnome image analyzer (Syngene, Cambridge, United Kingdom). The integrated analysis of pixels of the labeled bands in the membranes was measured using the Image J software (Image Processing and Analysis in Java, version 1.8.0, available for free download at https://imagej.nih.gov/ij/).

### Statistical analysis

Values were analyzed by the GraphPad Prism software package, v. 6.01 (GraphPad, San Diego, CA), and the level of significance was determinate using one-way analysis of variance (ANOVA) followed by Dunnett’s multiple comparisons test. Error bars show the standard error of the mean (SEM). Unpaired Student’s t-test was used to compare each treatment with the control. A p-value < 0.05 indicated statistical significance.

## Results

### Quality and reproducibility of venom

As shown in Additional file 1: Figure S1, HPLC demonstrated that there was no significant chemical difference between the two extracted PnV venom samples.

### The first PnV separation obtained three fractions (F1, F2 and F3)

The procedure for separation of the crude venom into fractions was performed by molecular mass, using molecular filters with nominal separation at 10 and 3 kDa, according to Santos et al. [18]. Three main fractions were obtained: F1 (low weight, less than 3 kDa), F2 (intermediate weight, between 3 and 10 kDa) and F3 (high weight, above 10 kDa). These fractions are still complex mixtures and were evaluated by the migration test to select those with the most significant effects on GB cells.

### Cell migration was delayed by treatment with F1 and F2, but not with F3

**Fig. 1:**
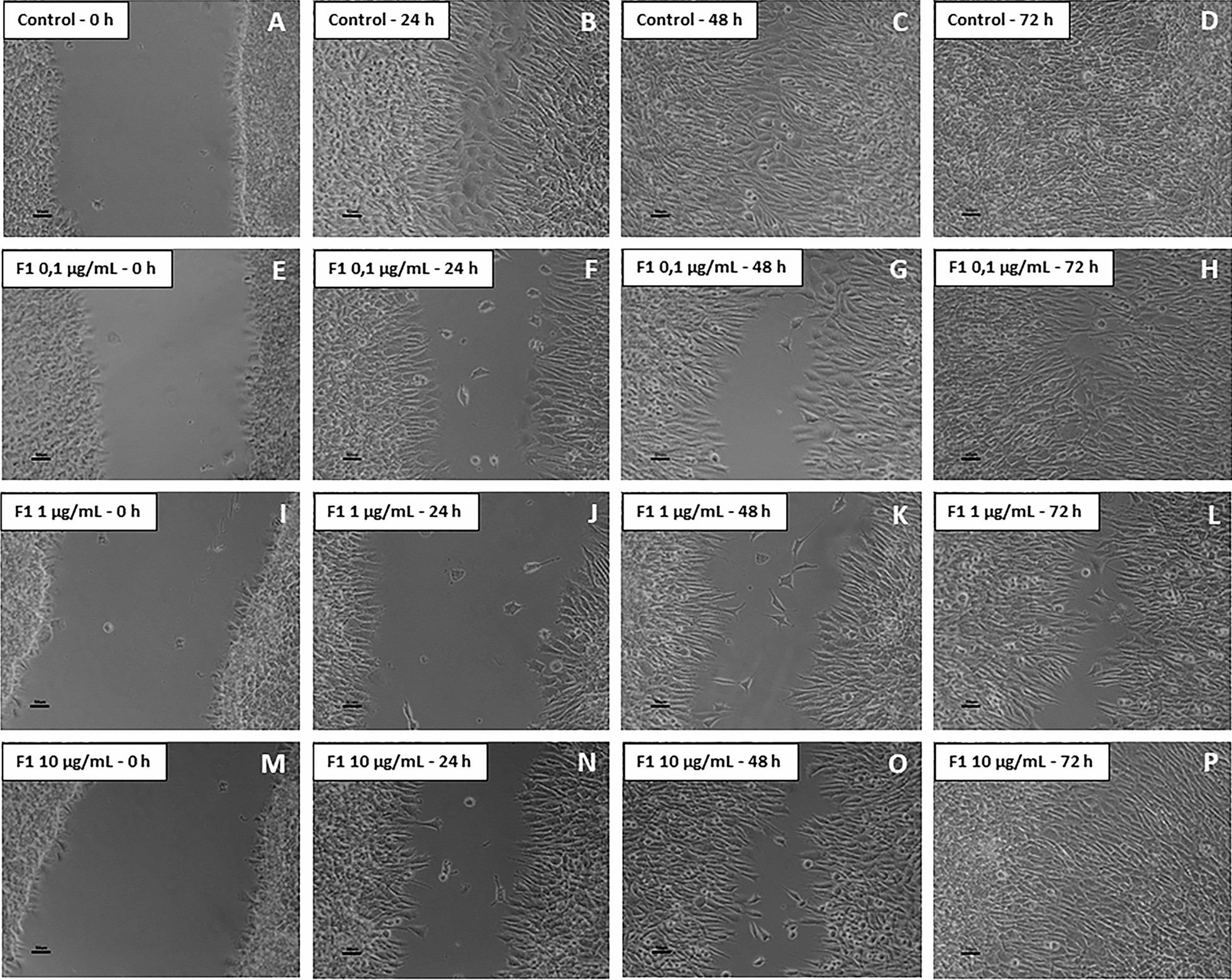
Migration assay by scratch-wound healing on F1-treated glioblastoma (NG97) cells. **a**–**d** Control (untreated cells); 72 h after the scratch, it was completely closed. **e**–**h** When exposed to F1 at 0.1 µg/ml, the cells showed a delay in the wound closure, filling it only after 72 h. **i**–**l** Cells treated with F1 (1.0 µg/ml), showed the most relevant delay in wound closure and did not fill the scratch even after 72 h. **m**–**p** F1 at 10 µg/ml also induced a delay in wound closure, but the cells could fill it after 72 h. Results of three independent experiments. Bars = 50 µm

**Fig. 2:**
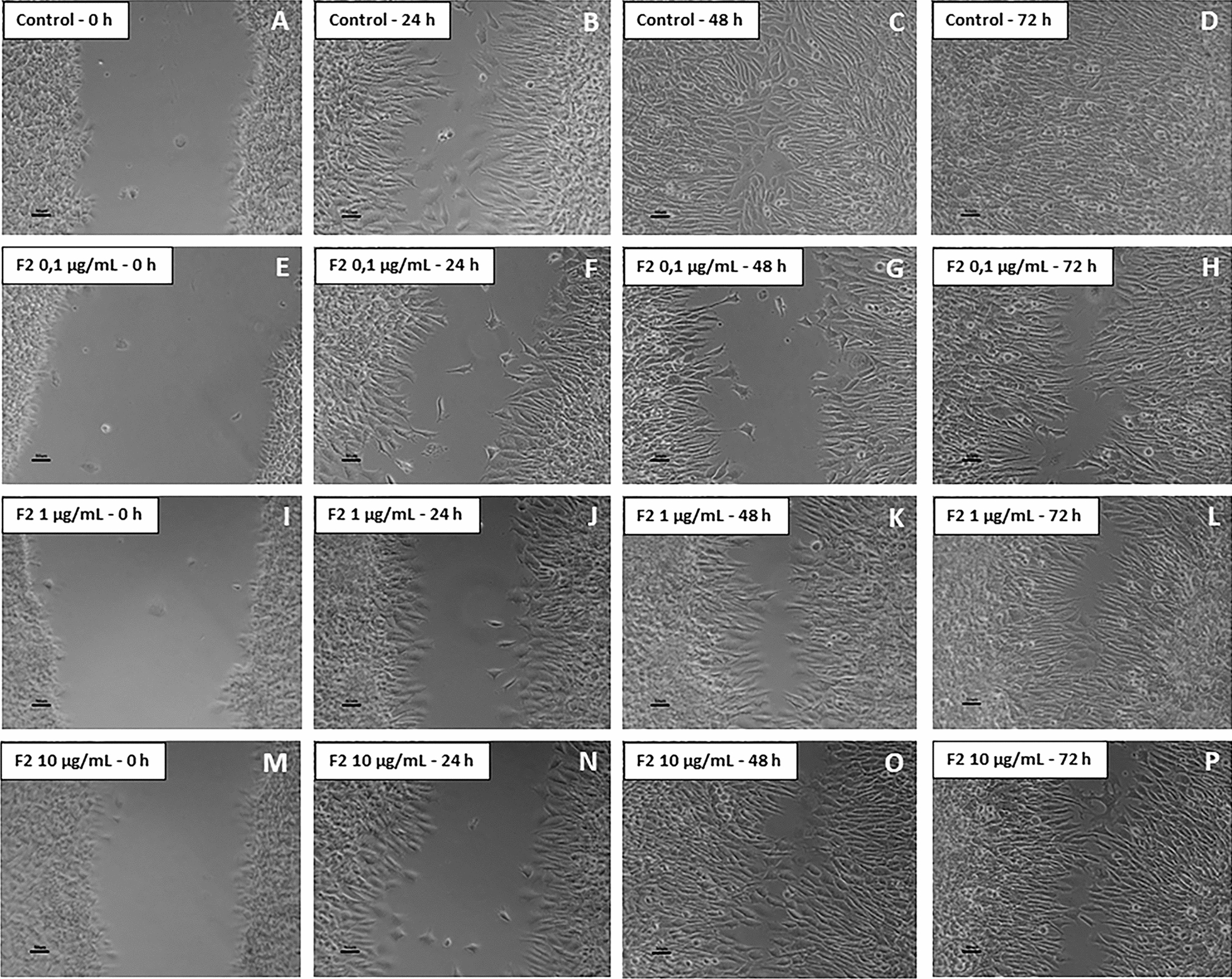
Migration assay by scratch-wound healing on F2-treated glioblastoma (NG97) cells. **a**–**d** Control (untreated cells); 72 h after the scratch, it was completely closed. When exposed to F2 at 0.1 µg/ml (**e**–**h**), 1 µg/ml (**i**–**l**) and 10 µg/ml (**m**–**p**), the cells showed a delay in the wound closure, being not able to filling it even after 72 h. Results of three independent experiments. Bars = 50 µm

**Fig. 3:**
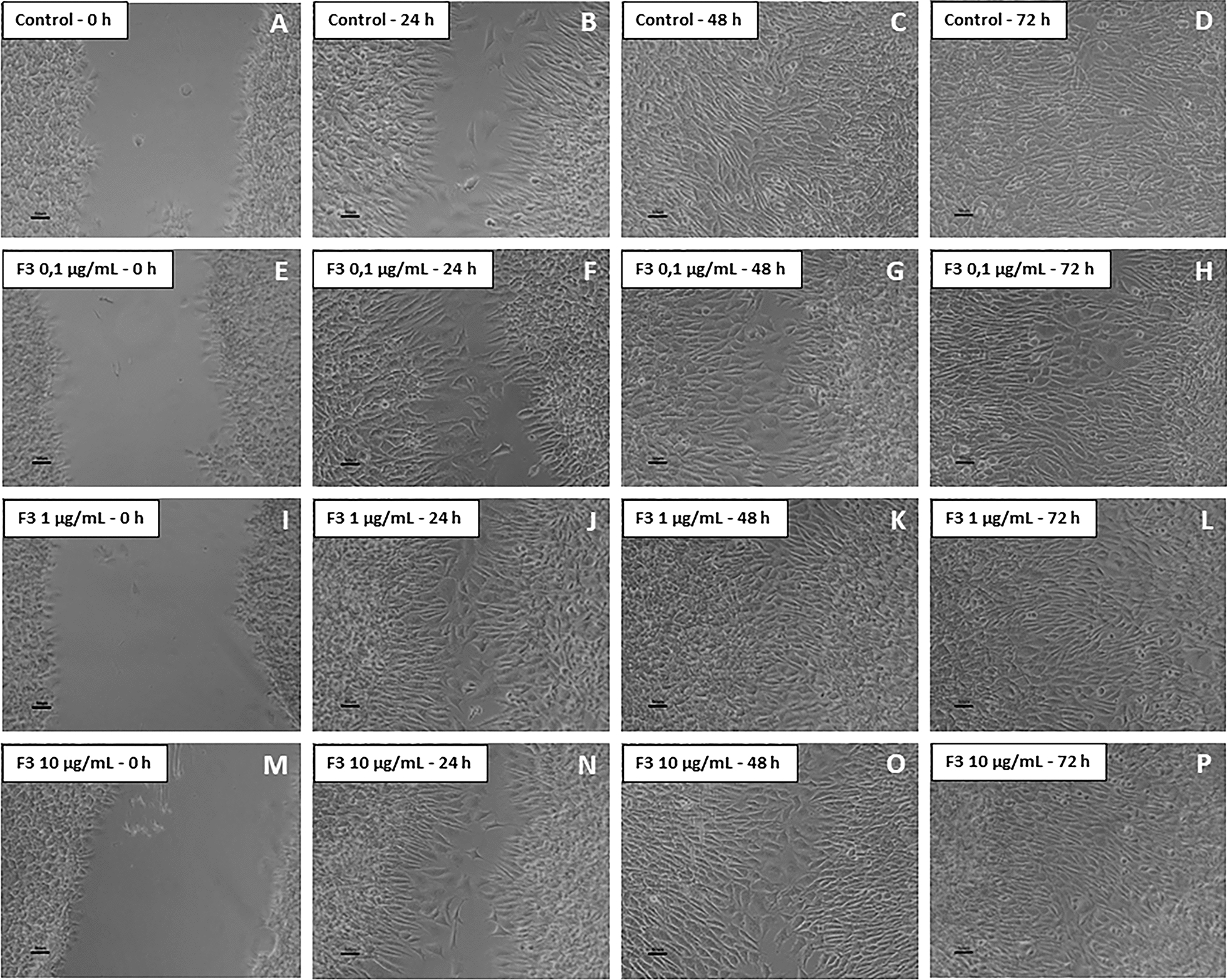
Migration assay by scratch-wound healing on F3-treated glioblastoma (NG97) cells. **a**–**d** Control (untreated cells); 72 h after the scratch, it was completely closed. No difference was observed in cells exposed to F3 at 0.1 (**e**–**h**), 1.0 (**i**–**l**) and 10 µg/ml (**m**–**p**) compared to control. Results of three independent experiments. Bars = 50 µm

The scratch-wound healing assay demonstrated that fractions F1 and F2 were effective in impairing GB cell migration (Figs. 1 and 2, respectively). Control cells completely filled the scratch after 72 h (Figs. 1a–d and 2a–d). On the other hand, F1-treated cells were delayed and could not migrate and fill the wound even after 72 h (Fig. 1e–p); the concentration of 1.0 µg/ml was more effective compared to 0.1 and 10 µg/ml (Fig. 1i–l). Similarly, cells incubated with F2 failed to migrate and did not fill the wound after 72 h (e–p of Fig. 2). On the other hand, F3-treated GB cells showed no difference compared to the control (Fig. 3). Considering these results, F1 and F2 (at 1.0 µg/ml) were chosen to continue the study and F3 was discarded.

### Proliferation and apoptosis necrosis assays with F1, F2 and F3

To analyze whether the fractions would affect proliferation or induce death of GB NG97 cells, CFSE and annexin/Pi assays were performed after 24 h of exposure to F1, F2 or F3. CFSE labeling showed that F1 and F2 did not induce increased cell proliferation, compared to the control. On the other hand, cells exposed to F3 showed a significant augment in proliferation (Fig. 41a–d). The annexin/Pi assay showed that the fractions did not induce significant necrosis or apoptosis of GB cells (Fig. 42a–g).

**Fig. 4:**
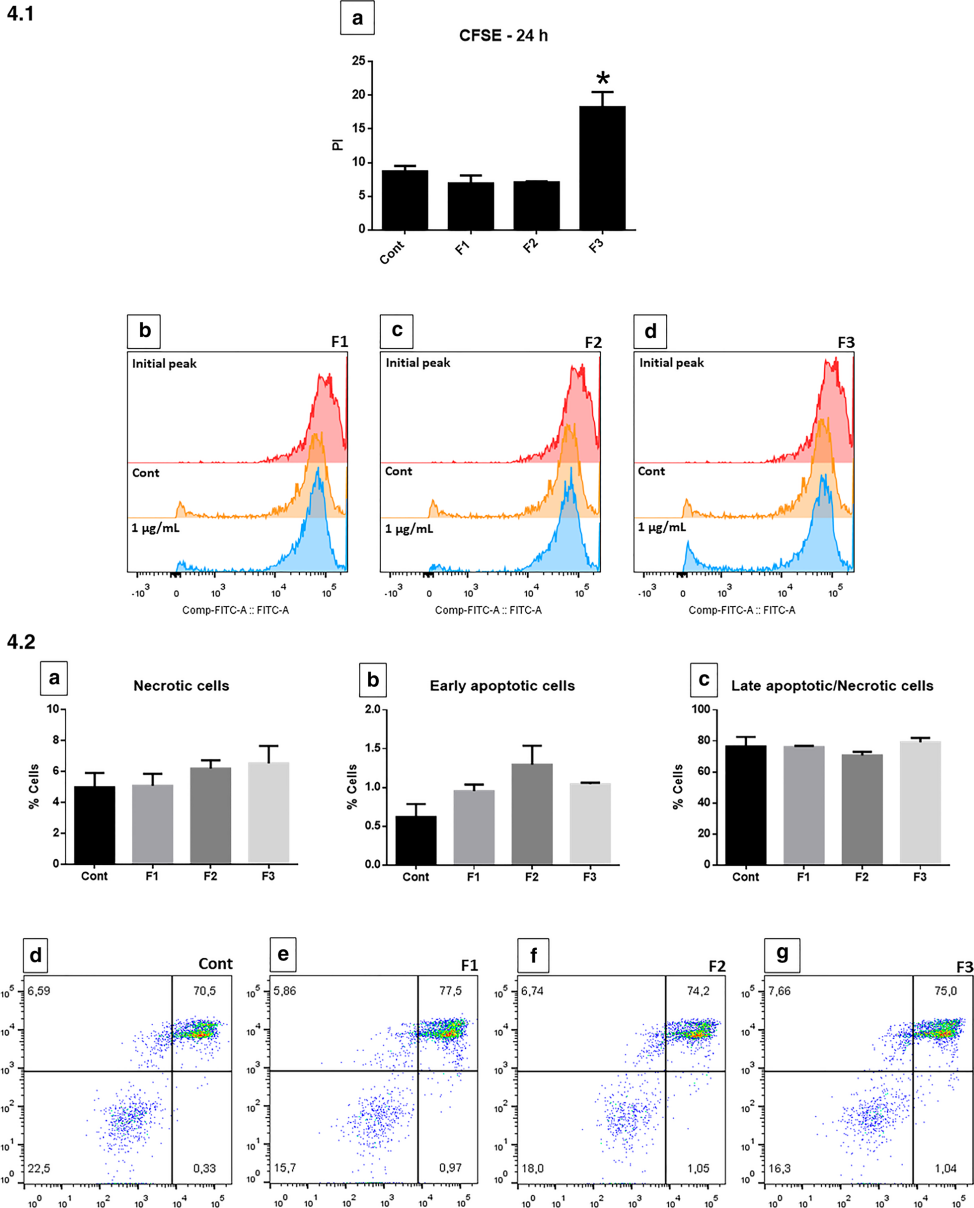
CFSE proliferation (4.1) and annexin/propidium iodide (Pi) (4.2) assays after 24 h of F1, F2 or F3. CFSE labeling showed that F1 and F2 (4.1. **b**, **c**) did not induce increased cell proliferation, compared to the control. Cells exposed to F3 showed a significant augment in proliferation (Fig. 4.1 **d**). The annexin/Pi assay showed that the fractions did not induce significant necrosis or apoptosis of GB cells (4.2 **a**–**g**). *P < 0.05, compared to control. Results of three independent experiments.

### Purification of F1 and F2 by HPLC, and MTT assay using subfractions

**Fig. 5:**
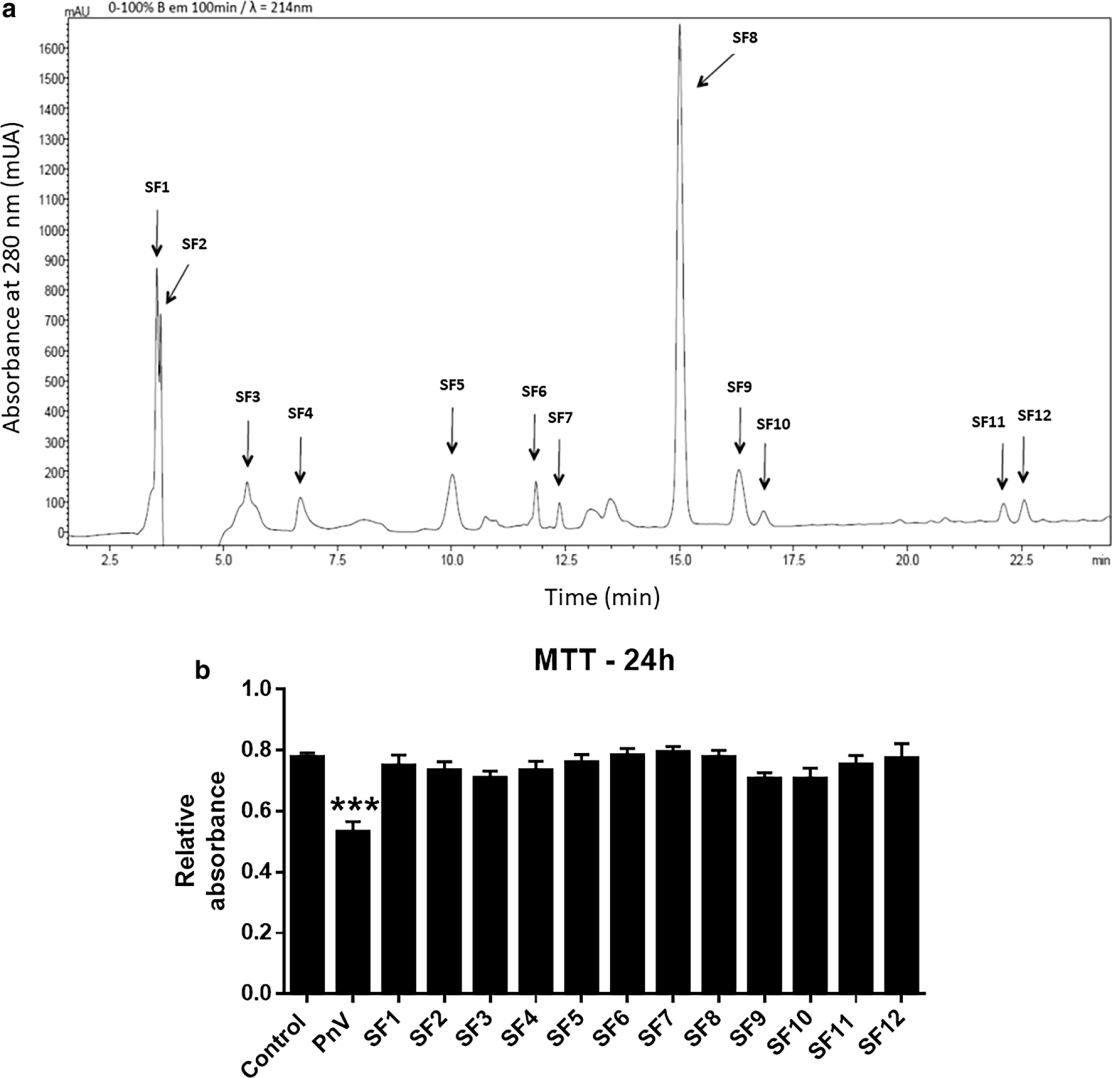
**a** Purification of F1 and F2 by high – pressure liquid chromatography (HPLC). This procedure generated twelve subfractions, named SF1 to SF12. **b** Cell viability assay (MTT) performed after 24 h of PnV and SF1-SF12 exposure. The subfractions (1.0 µg/ml) did not induce change in the viability of glioblastoma (NG97) cells viability. Only de crude venom (PnV) induced a decrease, compared to control. All toxins were used at 1.0 μg/ml. PnV (14 μg/ml) promoted a subtle decrease of cell viability (not significant). ***P < 0.001, compared to control. Results of three independent experiments

F1 and F2 were pooled and purified by HPLC. This procedure generated 12 subfractions, called SF1 through SF12 (Fig. 5a). These PnV-components were tested for effects on the ability of cells to fill the scratch in the wound healing assay, as reported below. In addition, to confirm that the observed effects on cell migration, invasiveness and adhesion were not due to a decrease in cell number by decreased cell survival, experiments were performed to test cell viability after exposure to PnV and SFs 1—12. The crude venom induced a significant decrease in cell survival, compared to control. However, no subfraction tested decreased GB cell viability after 24 h of exposure, at 1.0 µg/ml (Fig. 5b).

### SF1 and SF11-treated GB cells failed to migrate and fill the scratch; ROCK inhibition abolished the effect of both toxins

**Fig. 6:**
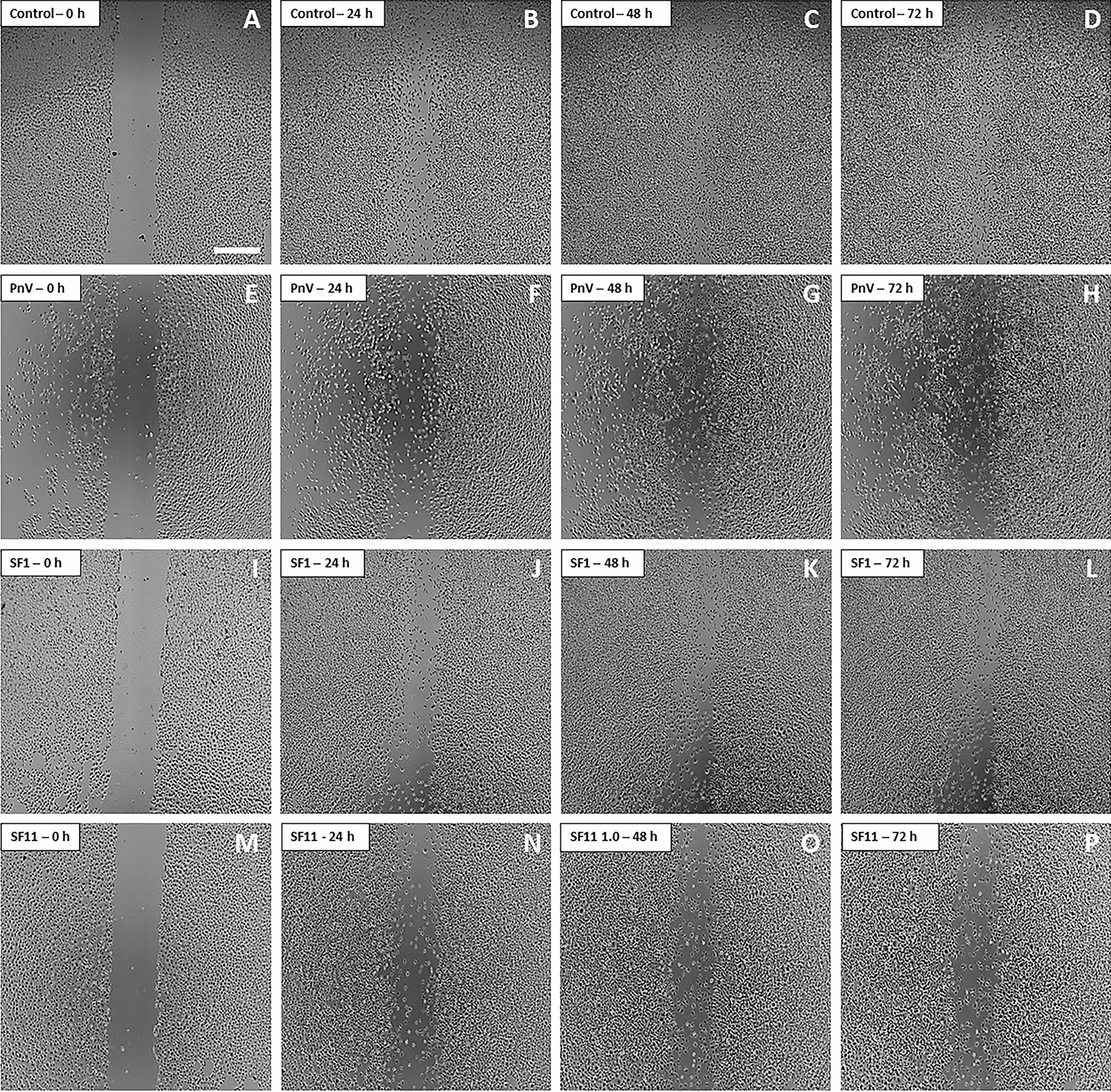
Migration assay by scratch-wound healing on SF1 – SF12-treated glioblastoma (NG97) cells. **a**–**d** Control Cells. The cells began to close the wound after 24 h and after 72 h it was completely closed. **e**–**h** Cells treated with PnV (14 µg/ml) showed a delay to fill the scratch, which was not closed until 72 h. **i**–**p** Cells incubated with SF1 and SF11 (1.0 µg/ml) also have failed to close the wound even after 72 h. Results of three independent experiments. Bars = 500 µm

A scratch-wound healing assay was performed with all 12 SFs (1.0 µg/ml) obtained from the separation of F1 and F2 by HPLC (Fig. 6). This screening revealed that while control cells (Fig. 6a–d) completely filled the scratch after 72 h, cells treated with SF1 (Fig. 5i–l) and SF11 (Fig. 6m–p) could not close the wound even after 72 h. On the other hand, the other toxins have intermediate effects (data not shown). Cells incubated with the crude venom (PnV), used as a control, also could not fill the scratch (Fig. 6e–h). Considering this screening, SF1 and SF11 were chosen to continue the study as the toxins with the best effects. Migration of untreated and SF11-treated cells was recorded for 72 h and the film is available in Additional files 2, 3: Movies S1 and S2.

**Fig. 7:**
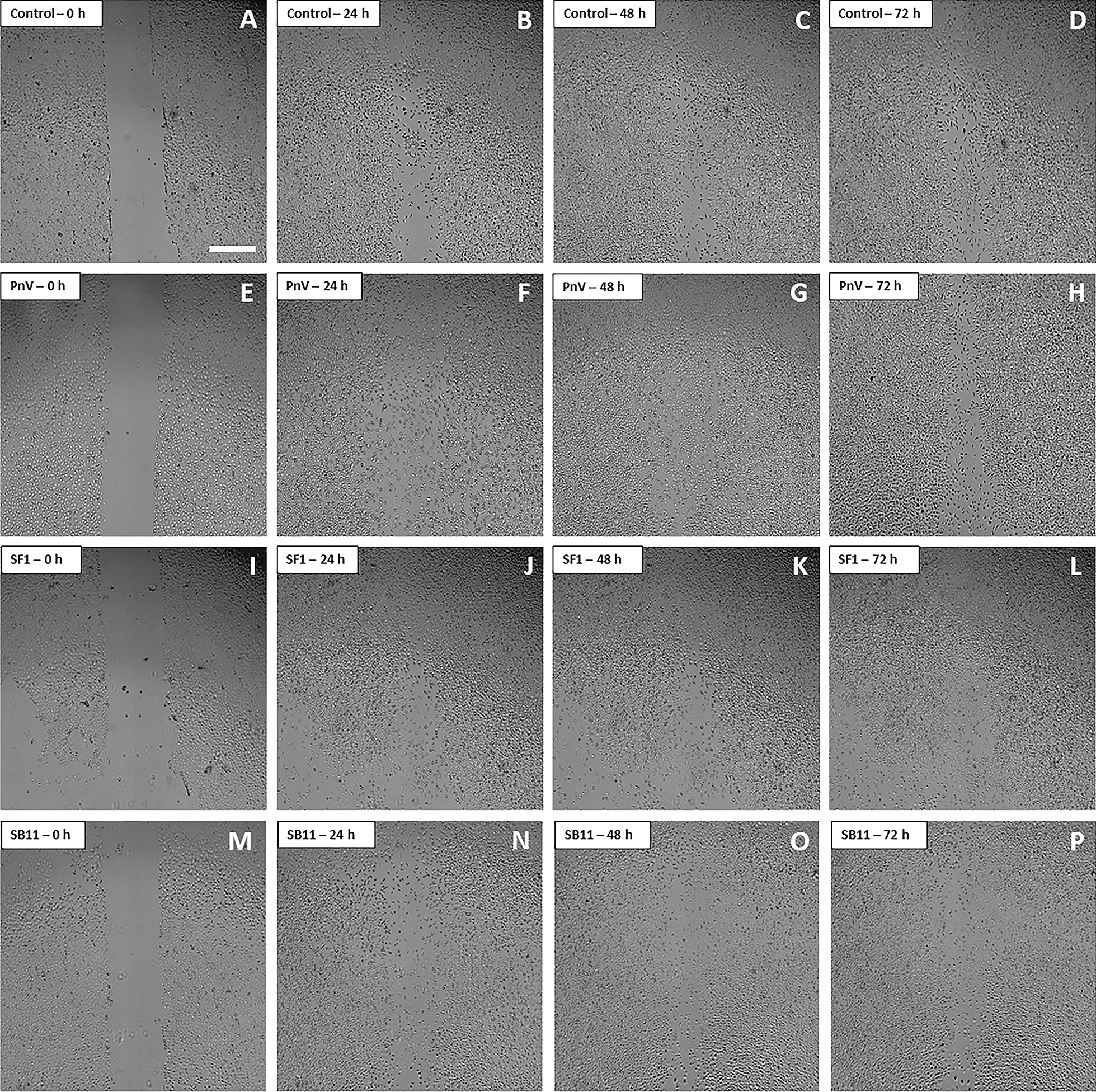
Migration assay by scratch-wound healing on PnV and SFs-treated glioblastoma (NG97) cells, with inhibition of ROCK. **a**–**d** Control Cells. The cells began to close the wound after 24 h and after 48 h it was completely closed. Cells treated with PnV (14 µg/ml) (**e**–**h**), SF1 (**i**–**l**) and SF11 (**m**–**p**), all at 1.0 µg/ml, showed a very similar behavior comparing to Control cells. Results of three independent experiments. Bars = 500 µm

To investigate the involvement of the RhoA-ROCK pathway, some wells were preincubated with Y-27632 (ROCK inhibitor) (Fig. 7). The result revealed that, when ROCK was inhibited, cells treated with SF1 and SF11 (Fig. 7i–l and m–p, respectively) were able to almost close the scratch after 72 h, showing only a subtle difficulty in migrating. Control cells (Fig. 7a–d), treated with ROCK inhibitor only, completely filled the scratch after 48 h. These results indicate that the RhoA-ROCK signaling may be involved in the effects of SFs on cell migration. Cells incubated with the crude venom (PnV) also could fill the scratch when ROCK was inhibited (Fig. 7e–h).

### Transwell assay showed that SF1 and SF11 strongly reduced GB cell invasion; The inhibition of ROCK did not change this effect

**Fig. 8:**
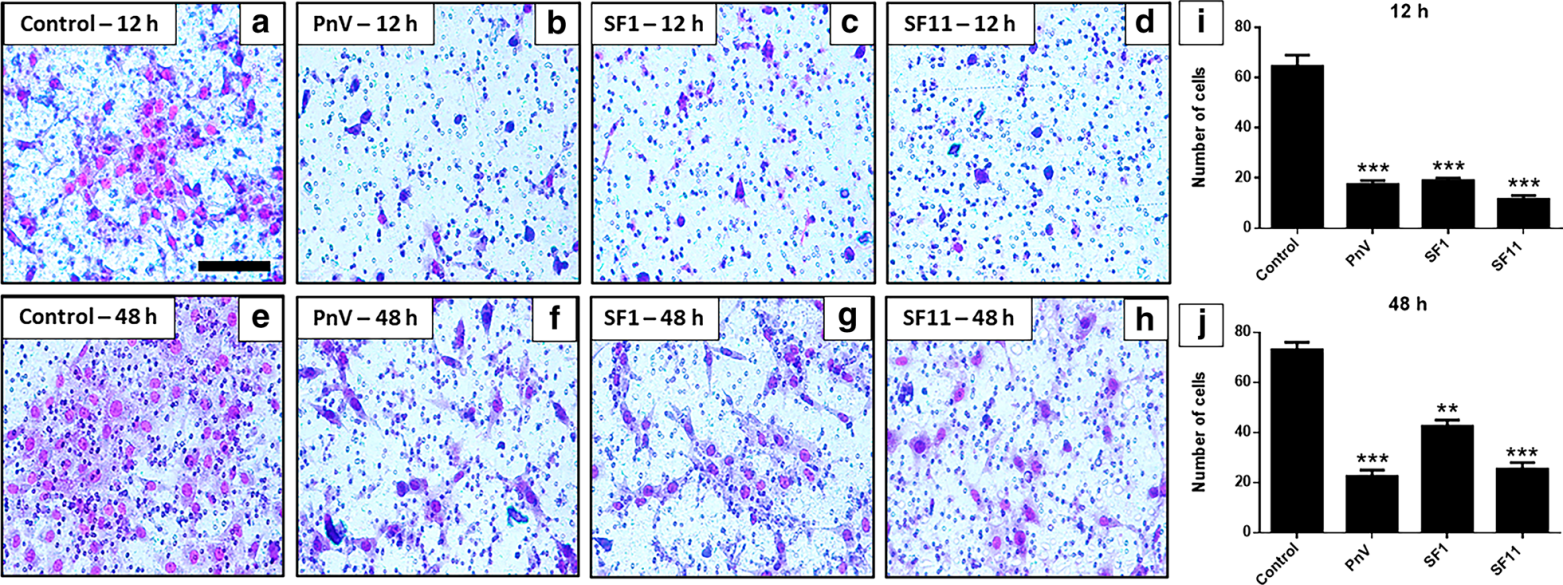
Transwell invasion test on glioblastoma (NG97) cells after 12 and 48 h of treatment. **a** and **e**–Cells maintained in medium (Control); **b** and **f**—Cells treated with PnV at 14 μg/ml. **c** and **g** Cells incubated with SF1 (1.0 μg/ml); **d** and **h**—Cells incubated with SF11 (1.0 μg/ml). Note that cells treated with PnV and SFs showed a significant reduction in the number of migrating cells (both 12 and 48 h), in comparison with the control. Graphs **i** and **j** present the statistical comparison, where **P < 0.01 and ***P < 0.001, compared to control. Results of three independent experiments. Bars = 100 µm

The transwell invasion assay showed that untreated (control) cells were able to migrate to the lower membrane surface after 12 h (Fig. 8a), while cells treated with PnV (14 μg/ml) migrated significantly less (Fig. 8b). SF1 and SF11 (1.0 μg/ml) induced a marked reduction in the number of migrant cells when compared to the control group (Fig. 8c, d). Similar results can be observed after 48 h of the treatments (Fig. 8e–h). Statistical data are shown in Graph I and J of Fig. 8.

**Fig. 9:**
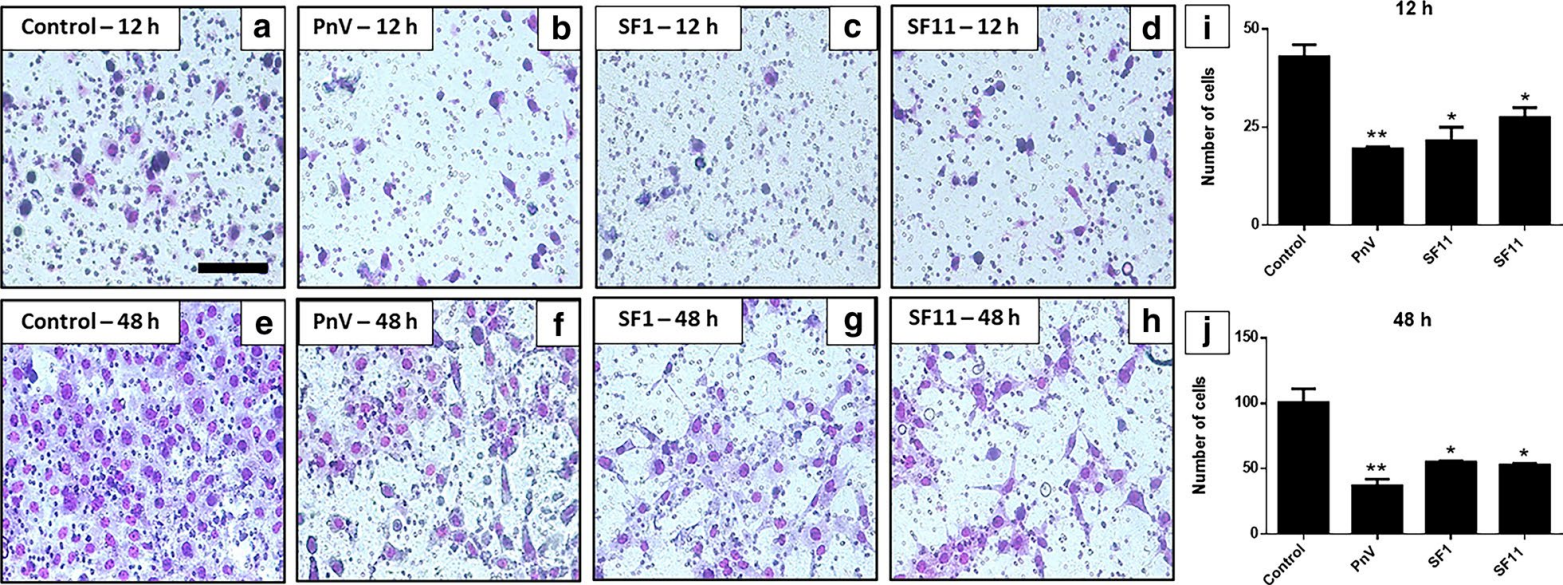
Transwell invasion test on glioblastoma (NG97) cells after 12 and 48 h of treatments. Cells received a ROCK inhibitor prior and during the treatments. **a** and **e**—Cells maintained in medium (Control); **B** and **f**—Cells treated with PnV at 14 μg/ml. **c** and **g** Cells incubated with SF1 (1.0 μg/ml); **d** and **h**—Cells treated with SF11 (1.0 μg/ml). Note that, even when ROCK was inhibited, cells treated with PnV, SF1 and SF11 showed a significantly reduction in the number of migrating cells (both 12 and 48 h) compared to Control. Graphs **i** and **j** present the statistical comparison, where *P < 0.05 and **P < 0.01, compared to control. Results of three independent experiments. Bars = 100 µm

When cells were incubated with ROCK inhibitor alone, they were able to migrate to the lower surface of the membrane after 12 h (Fig. 9a). Cells treated with PnV (14 μg/ml) migrated significantly less than cells incubated with the ROCK inhibitor (Fig. 9b). SF1 and SF11 (1.0 μg/ml) also showed a significant reduction in the number of migrant cells when compared to the Y-27632-Control group (Fig. 9c, d). Similar results were observed after 48 h of treatments (Fig. 9e–h). These data indicate that the RhoA-ROCK pathway is not involved with the effects of SFs components on cell invasion. Statistical data are shown in Graphs I and J of Fig. 9.

### Subfractions induced changes in cell morphology, as demonstrated by stress fibers labeling; ROCK inhibition abolished this effect

**Fig. 10:**
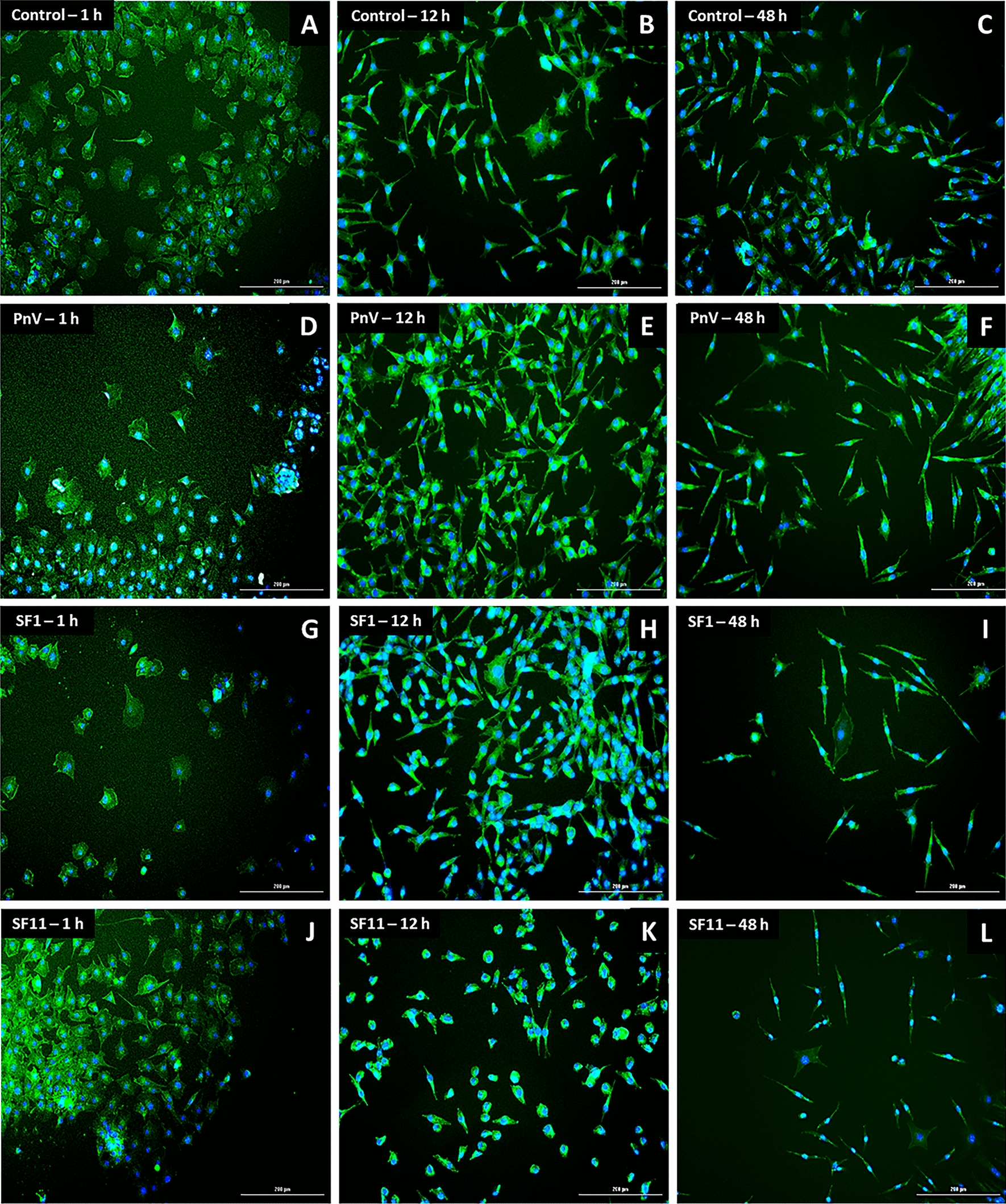
Phalloidin probe labelling on glioblastoma (NG97) cells 1, 12 and 48 h after treatments. Note that Control (untreated) cells (**a**–**c**) showed a morphological change over the time, becoming longer and thinner. PnV, SF1 and SF11 (**d**–**f**, **g**–**i** and **j**–**l**, respectively) induced a different morphology (arrows), with rounder cells at the beginning, which become more stellate over time. Results of three independent experiments. Bars = 200 µm

**Fig. 11:**
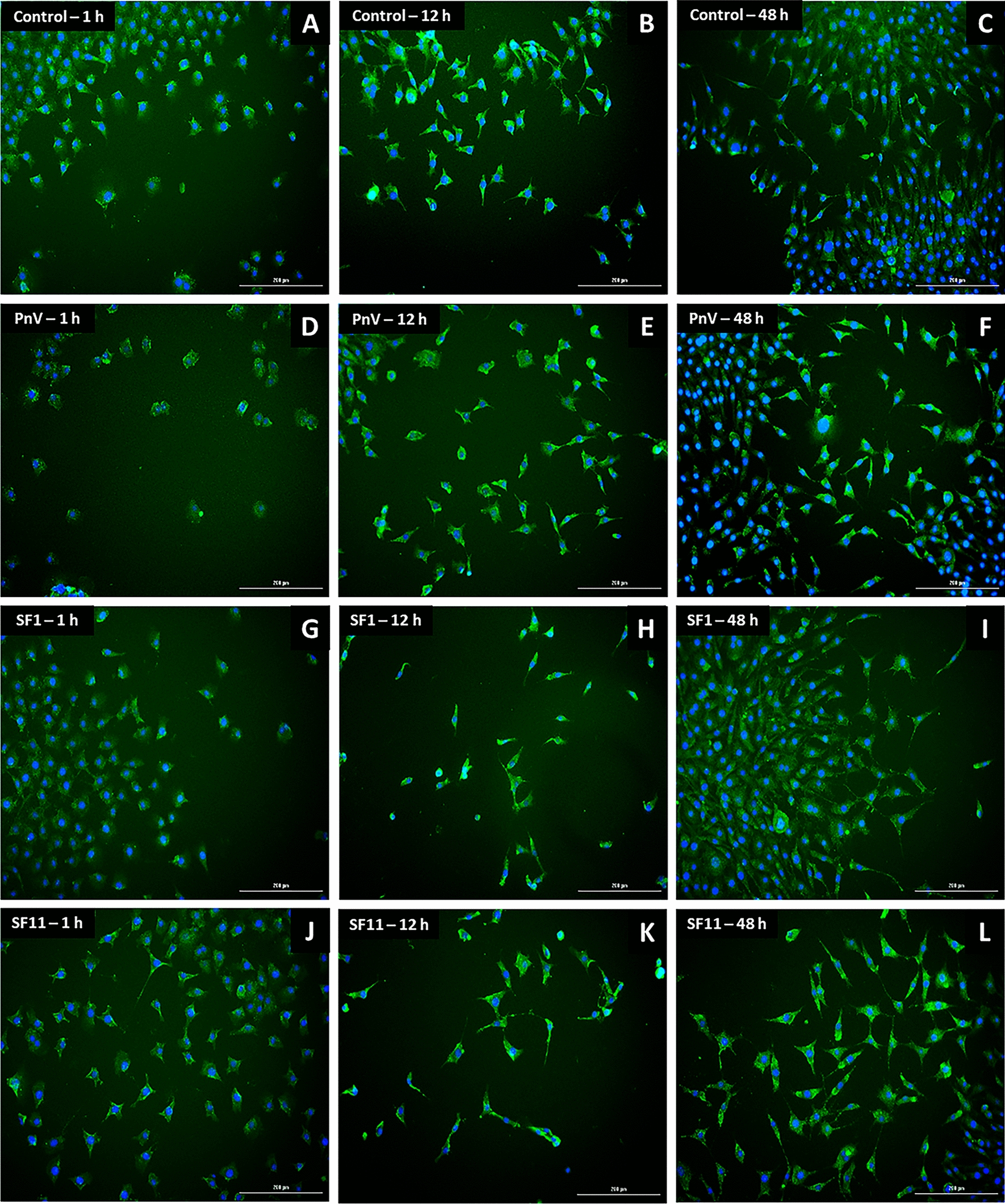
Phalloidin probe labelling on glioblastoma (NG97) cells (with ROCK inhibition) 1, 12 and 48 h after treatments. Note that Control (untreated cells; **a**–**c**) showed a morphological change over the time, becoming longer and thinner. PnV (**d**–**f**) and all toxins (SF1, **g**–**i** and SF11, **j**–**l**) had no effects, inducing the same morphological profile observed in Control cells. Results of three independent experiments. Bars = 200 µm

The labeling of F-actin (stress fibers) with a phalloidin probe was made to highlight the cytoskeleton, allowing the analysis of cell morphology (Figs. 10 and 11). Cells were followed over time and Control (untreated) showed subtle morphological alteration considering the time points (1, 12 and 48 h; see a–c of Figs. 10 and 11). Cells treated with PnV, SF1 and SF11, on the other hand, had a more circular/oval appearance at baseline (1 and 12 h), becoming thinner, bigger and wider than Control cells after 48 h of treatments (Fig. 10, d–l). However, when ROCK was inhibited (Fig. 11), this difference was not observed, and cells treated with PnV or SFs showed a similar morphological profile over time to the control. The cells were GFAP positive (Additional files 4, 5: Figures S2 and S3 – without and with ROCK inhibitor, respectively), confirming the astrocytic origin of the tumor.

### SF1 and SF11 decreased cell adhesion; when ROCK was inhibited, this effect was abolished

**Fig. 12:**
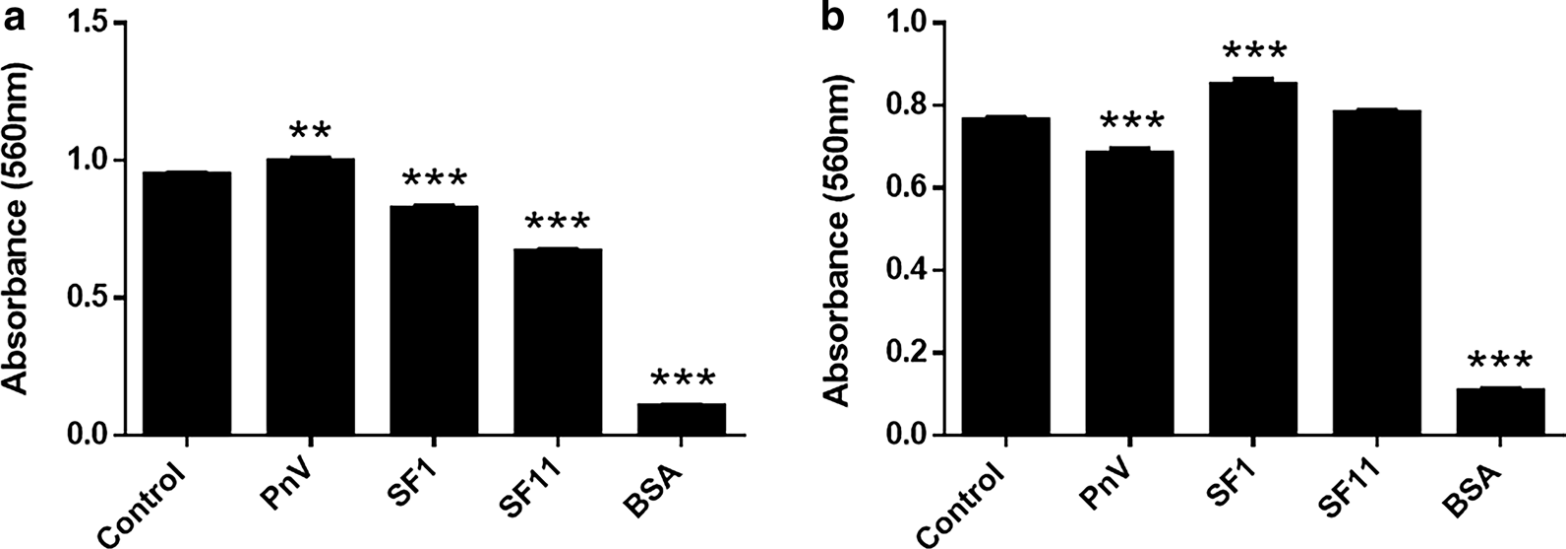
Cell Adhesion assay in glioblastoma (NG97) cells. **a** SF1 and SF11 significantly reduced cell adhesion, compared to Control cells. PnV induced an increase in the number of adherent cells. **b** Under ROCK inhibition, SF1 significantly increased cell adhesion and SF11 did not induce significant effect compared to Control cells; PnV induced a decrease in the number of adherent cells. Wells without fibronectin were used as a negative control and showed a minimum adhesion of cells (BSA wells). ***P < 0.001 and **P < 0.01, compared to control. Results of three independent experiments

The adhesion assay was performed by seeding the same number of cells from different groups in a 24-well plate coated with fibronectin. The assay showed that SF1 and SF11 significantly reduced cell adhesion compared to Control cells (Fig. 12a). Interestingly, cells treated with PnV showed a significantly increased adhesion, what is probably due to other components present in the crude venom. When ROCK was inhibited by Y-27632, the effect of SFs was abolished; SF1 increased cell adhesion and SF11 did not induce significant effect (Fig. 12b).

### SF1 and SF11 increased ROCK and Na^+^/K^+^-ATPase β2 (AMOG) levels

**Fig. 13:**
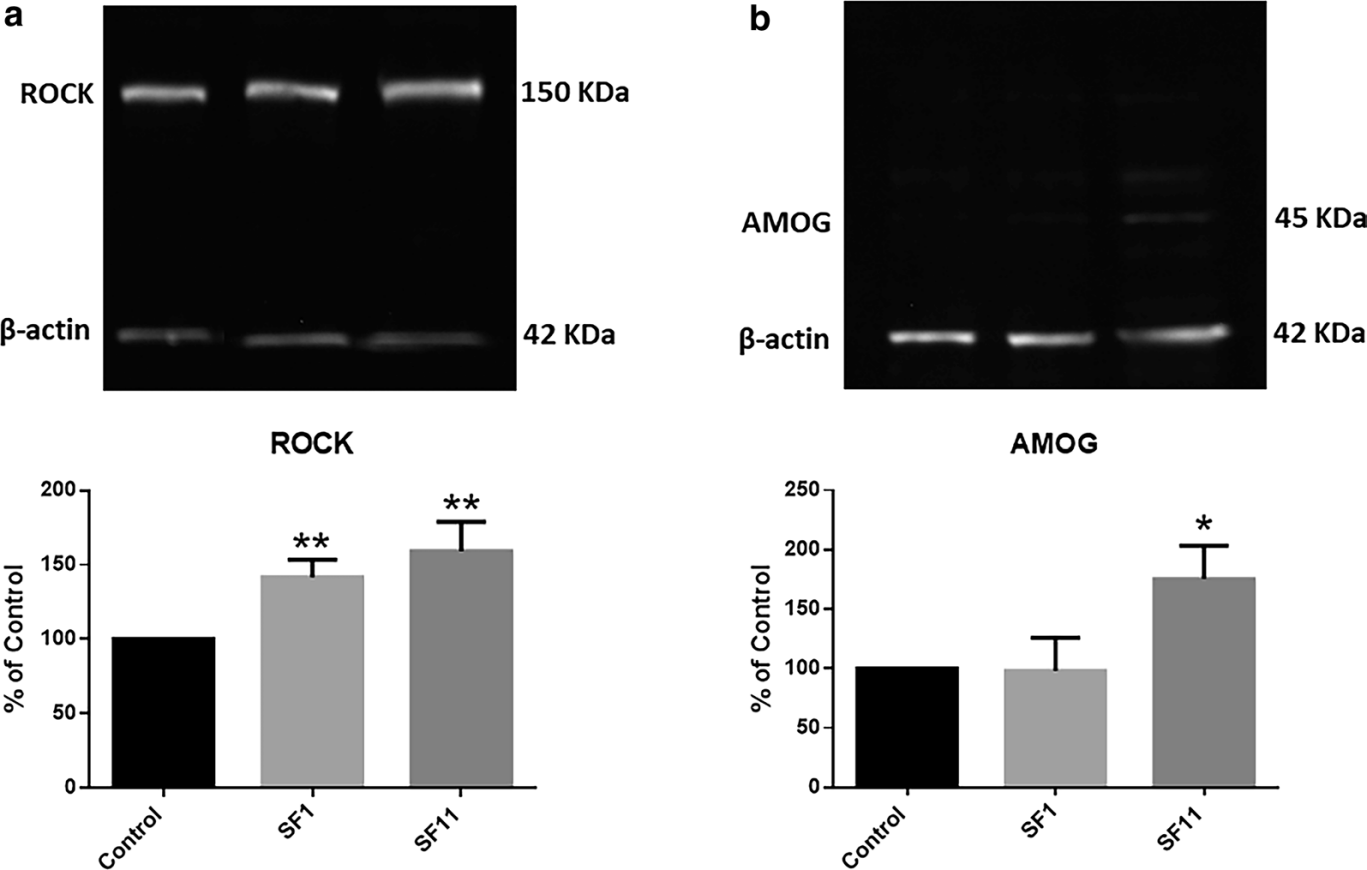
ROCK and Na^+^/K^+^-ATPase expression assessed by Western blotting. The expression of ROCK was significantly increased after 5 h of both treatments (SF1 and SF11) (**a**). The expression of Na^+^/K^+^-ATPase (AMOG) was significantly increased after 5 h of treatment with SF11 (**b**). *P < 0.05 and **P < 0.01, compared to the untreated control. Results of three independent experiments

To clarify the antineoplastic mechanism of SFs, western blotting was performed to evaluate ROCK and AMOG, as these proteins are involved in regulating cell migration. Both SF1 and SF11 significantly increased the levels of ROCK, compared to Control (untreated) (Fig. 13a), suggesting that RhoA-ROCK signaling is activated by these PnV components. In addition, AMOG (Na^+^/K^+^-ATPase β2 isoform) increased when GB cells were treated with SF11 (Fig. 13b). All original membranes are shown in Additional files 6, 7: Figures S4 and S5 (ROCK and AMOG, respectively).

## Discussion

High-grade gliomas, such as GB, are extremely aggressive brain tumors that often become rapidly fatal [24]. Surgical removal represents a standard treatment, increasing the average survival of the patient. However, GB cells have the ability to migrate and infiltrate the normal parenchyma, leading to the formation of recurrent tumors, frequently adjacent to the site of surgical extraction, being the main cause of treatment failure [5, 25]. Therefore, it is still necessary to develop new treatments for GB and to find molecules that decrease cell migration and invasion may improve prognosis after surgery.

Most cancer drugs used and under development interfere with cell proliferation, cycle and/or survival. Studies using venoms have also mainly identified molecules targeting these characteristics of tumorigenesis. For example, Gupta et al. [26] reported in vitro anti-proliferative and apoptogenic activity induced by *Heterometrus bengalensis* Koch (Scorpionidae) (Indian black scorpion) in human leukemic (U937 – histiocytic lymphoma and K562 – chronic myelogenous leukemia); The *Macrothele raveni* spider also induced apoptosis and cell cycle arrest in G0/G1 in human hepatocellular carcinoma [27]. However, molecules that target cell migration and invasiveness have been less explored.

Previous studies published by our group screened the antitumor effects of the *Phoneutria nigriventer* spider venom (PnV) on cell lines for proliferation, survival, cell cycle and migration, showing that the venom decreased the viability by inhibiting cell cycle and inducing death, and also delayed its migration [13, 18]. The venom significantly affected all tumor cell lines studied, although the non-glioma tumor cell (HeLa – human cervical tumor line) was less sensitive than human GB cell lines (NG97 and U-251). A clinically relevant point is that the venom had no cytotoxic effect on non-tumor cells (murine fibroblasts L929). In addition, a recently published study showed that systemic administration of PnV (100 µg/kg, i.p.) for 14 days induced a reduction or eradication of xenogeneic implanted tumor in mice. The study also showed that the systemic treatment with the venom improved the general condition of the animals, increasing body weight and survival, while did not decrease blood cells, but increased monocytes, indicating the low toxicity of the treatment [28]. Considering that venoms are a complex mixture of molecules, in the present study we proceed with the investigation, working with isolated PnV molecules; Fractions (F1, F2 and F3) and subfractions (SFs) were tested and two SFs (SF1 and SF11—obtained from F1 and F2 together; F3 did not have effect on migration and was discarded) in a high degree of purification were identified as demonstrating the greatest effective action on migration, morphology, invasiveness and adhesion of GB cells. As a control parameter, to check if other effects could be interfering in the interpretation of the migration results, it was demonstrated here that the fractions F1 and F2 do not induce tumor cell proliferation and death (apoptosis or necrosis). F3, on the other hand, induced significant proliferation of GB cells. In addition, no SF induced decrease in cells viability.

SF1 and SF11 delayed the migration of GB cells in the scratch wound healing assay. Cell migration is a highly orchestrated mechanism and, under deregulation, actively contributes to metastasis [29]; Ion channel activities are directly linked tumor cell migration [30, 31]. Ion transporters play key roles in cancer cell migration in general and in GB in particular. For example, K^+^ channel stimulation increases migration and infiltration of GB cells in vivo [32]. Several studies have shown that PnV is composed of biologically active molecules. Some of these toxins have been isolated and characterized biochemically/pharmacologically and it is well established that several of these toxins block Ca^2+^ and K^+^ channels or delay Na^+^ channels inactivation [33, 34]. P/Q- and N-voltage-gated Ca^2+^ channel (VGCC) blockers derived from *P. nigriventer* venom have previously been shown to have anti proliferative effects, impairing tumor progression [35]. Also, Rapôso et al. [17] showed that this venom evoked Ca^2+^ transients from intracellular Ca^2+^ stores in astrocytes. Directional cell migration involves repeated cycles of protrusion at the front and retraction at the back of the cell [36–38]. Ca^2+^ is widely regarded as an important coordinator of these events [39–41]. Cell adhesion is another important phenotypic feature during GB progression and is also influenced by Ca^2+^ channel activity [28]. The detachment of cells from the primary glioma tumor mass comprises several events involving the destabilization and disorganization of cell–cell adhesion, as well as the loss of expression of neural cell adhesion molecules [42]. SF1 and SF11 may have channel blockers that contribute to the anti-migration effects, and this will be confirmed in a further study.

The Na^+^/K^+^-ATPase is overexpressed in a majority of GBs compared to normal brain tissues; This pump appears to be related to GB cell migration and invasion, rapidly adjusting its shape and volume as it invades the cerebral parenchyma [43]. Binding to the α1 subunit of Na^+^/K^+^-ATPase, UNBS1450 (a cardiac steroid) impairs the migration of human GB U373-MG cells through a disorganization of the actin cytoskeleton [44]. In addition, several isoforms of the Na^+^/K^+^-ATPase β-subunit have been shown to regulate cell adhesion, particularly in the context of cancer progression [45, 46]. The β2 isoform, also known as the adhesion molecule on glia (AMOG), plays a role in CNS cell adhesion [47, 48]. Although AMOG is highly expressed in normal adult CNS, evidence suggests that it can be downregulated or lost in most GBs [49]. Loss of AMOG has been implicated in glioma invasion and migration, while evidence suggests that AMOG expression in GB inhibits its invasion [50]. An earlier study from our group showed that PnV affects astrocytes, inducing profound changes in their morphology and cytoskeleton. Astrocytes showed altered actin filament structure after PnV exposure and the venom also increased Na^+^/K^+^-ATPase expression [17]. These results lead to the hypothesis that this pump, specifically AMOG, could be involved in the effect of SF1 and SF11 on GB cell migration and invasion. Indeed, it was demonstrated in this work that SF11 (but not SF1) induced a significant increase in AMOG expression, suggesting that this pump may be involved in the anti-invasive mechanism of these components.

Although AMOG is an important regulator of invasiveness, migration can be modulated by other mechanisms, including a Rho-dependent cascade. The Rho pathway is believed to induce adhesions focused on stimulating contraction through MLC phosphorylation [51]. This effect is mediated by the effector Rho-associated serine/threonine kinase ROCK [52]. Rho activates ROCK, which elevates MLC phosphorylation, increasing myosin activation [53]. In addition, normal stress fiber formation involves ROCK signaling [54]. Both SF1 and SF11 increased ROCK levels, suggesting that their components activated this pathway. In addition, inhibition of ROCK by Y-27632 abolished most effects of SFs.

Studies show that RhoA-ROCK signaling and consequent modulation of the cytoskeleton in cancer cells may be involved in regulating cell migration and metastasis [55]. Nutt et al. [56] showed that Rho-GTPases genes are highly correlated with glioblastoma. RhoA and RhoB expression was significantly reduced in astrocytic tumors and their levels were inversely proportional to tumor grade, ie, lower expression was associated with a higher grade and therefore a more aggressive neoplasm [57]. It has been shown that Rho is rapidly activated following the fixation of malignant astrocytoma cells on the substrate and that Rho activation is responsible for the morphological changes of these fixed cells [58]. Glioblastoma cells with increased RhoA activity are characterized by impaired cell migration due to the induction of profound morphological changes, including actin reorganization into stress fibers and the induction of focal adhesions [59]. These changes, mainly related to RhoA activity, make the cells immobile [60]. Furthermore, in human GB cells, ROCK activation decreases cell mobility [61], while ROCK inhibition results in increased migration [62, 63].

Therefore, as SFs increased ROCK expression and considering that the inhibition of this RhoA effector abolished the effects of SFs on GB cell migration, morphology and adhesion, it is clear that ROCK is involved in the mechanism of these components. Interestingly, transwell invasion was the only effect not abolished by ROCK inhibition. It has been shown that GB cells with AMOG expression restored again by an overexpression vector exhibited a drastically reduced invasion, but there was no difference in migration or proliferation compared to control cells [47]. Probably the mechanism behind the effect of SFs on GB cell invasion is prominently regulated by AMOG.

The present data began to clarify the effects and mechanisms of purified PnV toxins on the migration of tumor cells. The effects of the two SFs (SF1 and SF11) seem to be very similar considering the parameters observed in this study. Regarding the mechanisms, SF1 apparently involves ROCK while SF11 involves ROCK and AMOG; however, these and other mechanisms may be involved and will be clarified in further studies. In addition, in an ongoing study, the effect of SF1 and SF11 is being characterized in GB cells collected from patients and the response will be correlated to the molecular profile of the tumor. This will guide individual treatment, predicting tumors responsive to molecules and also will give light on the mechanisms of the toxins. In addition, the effects of toxins on patients' blood cells are being characterized to assess safety aspects in the use of these molecules as systemic therapy for the treatment of GB.

## Conclusion

In conclusion, this study demonstrated that components present in purified PnV subfractions (SF1 and SF11) decreased migration and invasion, which is probably a consequence of impaired cell morphology/cytoskeleton and adhesion. The mechanisms behind these effects involve Na^+^/K^+^-ATPase β2 (AMOG) and RhoA-ROCK signaling, considering that the effects were decreased or abolished when ROCK was inhibited and the expression of ROCK and AMOG where increased by PnV and or SFs. Other studies are being conducted to characterize and synthetize the molecules present in SF1 and SF11. This paper is the third in a series of studies focused on the development of a new pharmacological formulation for the treatment of glioblastoma.

## Supplementary information

**Additional file 1: Figure S1**. The PnV profile obtained by high – pressure liquid chromatography (HPLC) showed that there were no relevant differences between the two pooled venom samples used in this work.

**Additional file 2: Movie S1.** Glioblastoma (NG97) cell migration recording for 72 h after scratching; Cells received no treatment (control cells).

**Additional file 3: Movie S2.** Glioblastoma (NG97) cell migration recording for 72 h after scratching; Cells were exposed to SF11.

**Additional file 4: Figure S2.** GFAP immunolabeling on glioblastoma (NG97) cells 1, 12 and 48 h after treatments. This result shows that the cells keep the phenotype from astrocytes origin. In addition, this intermediate filament labeling confirms the morphological alterations induced by the PnV, SF1 and SF11 (D – F, G – I and J – L, respectively), demonstrated also by actin filament labelling by phalloidin probe. Cells were rounder at the beginning, which become more stellate over time. Results of three independent experiments. Bars = 200 µm.

**Additional file 5: Figure S3.** GFAP immunolabeling on glioblastoma (NG97) cells (with ROCK inhibition) 1, 12 and 48 h after treatments. This result shows that the cells keep the phenotype from astrocytes origin. In addition, this intermediate filament labeling confirms that the toxins (SF1, G-I and SF11, J-L) had no effects changing morphology, demonstrated also by actin filament labelling by phalloidin probe. Cells were rounder at the beginning, which become more stellate over time. Results of three independent experiments. Bars = 200 µm.

**Additional file 6: Figure S4.** Images of all original western blotting membranes of ROCK immunolabeling. 1 = Control, 2 = SF1, 3 = SF11.

**Additional file 7: Figure S5.** Images of all original western blotting membranes of AMOG immunolabeling. 1 = Control, 2 = SF1, 3 = SF11.

## Data Availability

The datasets used and/or analyzed during the current study are available from the corresponding author on reasonable request.

## Abbreviations

AMOG: Glial adhesion molecule
ANOVA: One-way analysis of variance
BBB: Blood–brain barrier
BSA: Bovine serum albumin
CNS: Central nervous system
DMSO: Dimethyl sulfoxide
F: Fractions
FBS: Fetal bovine serum
FDA: Food and Drug Administration
GB: Glioblastoma
GFAP: Glial fibrillary acidic protein
HPLC: High‐pressure liquid chromatography
HRP: Horseradish peroxidase
IMDM: Iscove’s modified Dulbecco’s medium
MTT: Thiazolyl Blue Tetrazolium Bromide
PAGE: Polyacrylamide gel electrophoresis
PBS: Phosphate buffered saline
PnV: *Phoneutria nigriventer* Spider venom
ROCK: Rho-associated protein kinase
SDS: Sodium dodecyl sulfate
SEM: Standard error of the mean
SF: Subfraction
TBS: Tris-buffered saline
TFA: Trifluoroacetic acid
