# Supplementary figures and images for: Spider venom components decrease glioblastoma cell migration and invasion through RhoA-ROCK and Na+/K+-ATPase β2: potential molecular entities to treat invasive brain cancer
Source: Cancer Cell Int. 2020 Dec 17;20:576. doi: 10.1186/s12935-020-01643-8 (PMC7745393; doi:10.1186/s12935-020-01643-8)

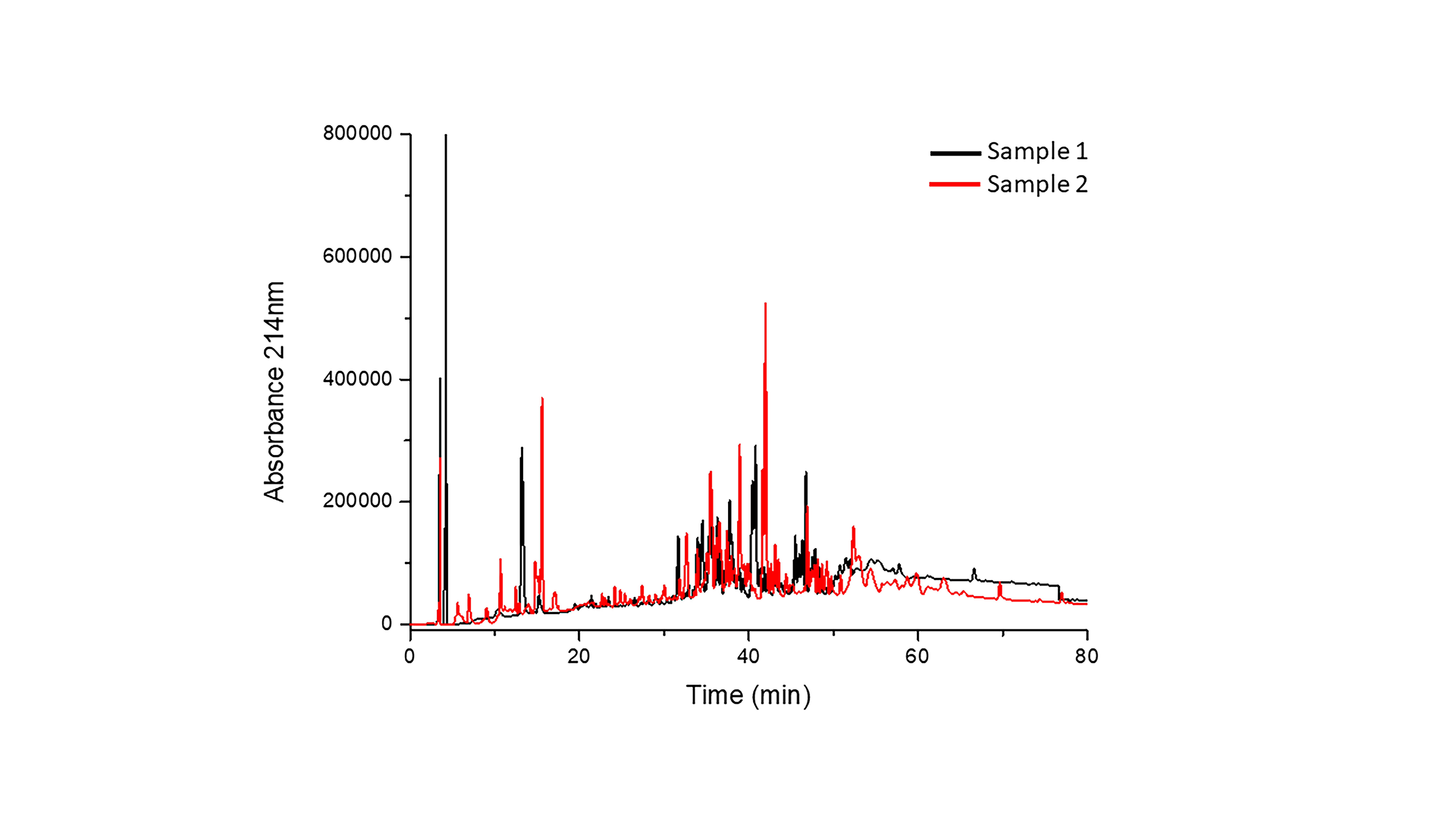

Supplement: Supplementary file 1 — Additional file 1: Figure S1. The PnV profile obtained by high – pressure liquid chromatography (HPLC) showed that there were no relevant differences between the two pooled venom samples used in this work. [file 12935_2020_1643_MOESM1_ESM.tif]
